# A Healthy Conversation Skills intervention to support changes to physical activity and dietary behaviours in community-dwelling older adults during the COVID-19 pandemic

**DOI:** 10.1177/17579139241262657

**Published:** 2024-08-01

**Authors:** J Zhang, I Bloom, LD Westbury, G Bevilacqua, KA Ward, M Barker, W Lawrence, C Cooper, EM Dennison

**Affiliations:** MRC Lifecourse Epidemiology Centre, University of Southampton, UK; NIHR Southampton Biomedical Research Centre, University of Southampton and University Hospital Southampton NHS Foundation Trust, UK; MRC Lifecourse Epidemiology Centre, University of Southampton, UK; NIHR Southampton Biomedical Research Centre, University of Southampton and University Hospital Southampton NHS Foundation Trust, UK; MRC Lifecourse Epidemiology Centre, University of Southampton, UK; MRC Lifecourse Epidemiology Centre, University of Southampton, UK; MRC Lifecourse Epidemiology Centre, University of Southampton, UK; NIHR Southampton Biomedical Research Centre, University of Southampton and University Hospital Southampton NHS Foundation Trust, UK; MRC Lifecourse Epidemiology Centre, University of Southampton, UK; NIHR Southampton Biomedical Research Centre, University of Southampton and University Hospital Southampton NHS Foundation Trust, UK; MRC Lifecourse Epidemiology Centre, University of Southampton, UK; NIHR Southampton Biomedical Research Centre, University of Southampton and University Hospital Southampton NHS Foundation Trust, UK; MRC Lifecourse Epidemiology Centre, University of Southampton, UK; NIHR Oxford Biomedical Research Centre, University of Oxford, UK; MRC Lifecourse Epidemiology Centre, University of Southampton, Southampton General Hospital, Tremona Road, Southampton SO16 6YD, UK

**Keywords:** behaviour change, diet, Healthy Conversation Skills, older adults, physical activity

## Abstract

**Aims:**

Physical activity (PA) and nutrition are important determinants of health in late adulthood. However, low levels of PA and poor nutrition are common in older adults and have become more prevalent during the COVID-19 pandemic. We hypothesised that Healthy Conversation Skills could be used to support health behaviour changes beneficial for health in older adults and thus conducted a study nested within the UK Hertfordshire Cohort Study.

**Methods:**

Between November 2019 and March 2020, 176 participants were visited at home. A trained researcher administered a questionnaire and undertook anthropometric and physical performance tests. A total of 89 participants were randomised to the control group and received a healthy living leaflet; 87 participants in the intervention group were interviewed using Healthy Conversation Skills at the initial visit with follow-up telephone calls at 1, 3, 6 and 9 months. Follow-up at 1 year by postal questionnaire assessed change in PA and diet. In total, 155 participants (79 control and 76 intervention) completed the baseline and 1-year follow-up.

**Results:**

At baseline, median (lower quartile, upper quartile) age (years) was 83.1 (81.5, 85.5) and median PA time (min/day) from walking, cycling and sports was 30.0 (15.0, 60.0). In total, 95% of participants completed the intervention; the total response rate for postal questionnaires was 94%. There were no statistically significant differences in outcomes between the trial arms. In women, there was a tendency for greater increases in diet quality in the intervention group compared to the control group (*p* = 0.075), while among men, there was a tendency for reduced decline in self-reported physical function in the intervention group compared to the control group (*p* = 0.081).

**Conclusion:**

We have shown that it is viable to utilise Healthy Conversation Skills via telephone to promote healthier lifestyles in older adults. Larger appropriately powered studies to determine the efficacy of such an intervention are now warranted.

## Introduction

The importance of physical activity (PA) and nutrition for human health has been widely endorsed especially in older adults.^1–3^ The 2015 World Report on Ageing and Health, published by the World Health Organisation (WHO), highlighted PA and adequate nutrition as key components to the concept of ‘healthy ageing’ and their importance in reducing age-related burden of disease and functional disability.^3,4^ However, PA levels are typically low in older adults: results from the 2021 Health Survey for England showed that only 10% of adults aged ⩾75 years met both the aerobic and the muscle-strengthening UK PA guidelines.^5^ In the latest update of the UK Chief Medical Officers’ Physical Activity Guidelines, there was a shift in messaging from stating that a specific amount of PA is required for better health to a recommendation that any increase in PA is beneficial, which it was hoped may encourage more PA participation in the older population.^2^ Likewise, poor quality of diet appears to be common in older populations, including within the UK.^6–8^ Studies have shown inadequate dietary intakes of some nutrients such as protein and key micronutrients among substantial proportions of older people in the UK.^9–11^ These unfavourable health behaviours, poor diet and low PA levels, often co-occur in older people.^12^

The coronavirus disease (COVID-19) was declared a pandemic by the WHO in March 2020, and subsequent restrictive measures introduced in many countries to reduce the transmission of the disease may have impacted negatively on daily routines and lifestyle. Many older adults were classified as ‘vulnerable’ in the UK, due to their advancing years and high probability of comorbidities. Those in the ‘vulnerable’ category were advised to remain shielded and only leave their homes when necessary, such as to seek medical attention. Therefore, any negative impact of the COVID-19 pandemic on lifestyle was felt acutely in older adults.^13,14^ Hence, research that considers how to prevent further decline in PA or diet quality, and even reverse such changes, is now urgently required.

This study aimed to assess the use of a participant-led behavioural intervention which included the use of Healthy Conversation Skills with community-dwelling older adults. Findings from our previous qualitative research in the Hertfordshire Cohort Study, a group of community-dwelling older adults, have indicated the potential for interventions that address psychosocial aspects, such as motivation, self-efficacy and social engagement, to promote dietary changes and PA participation.^15,16^ Furthermore, a recent review of healthy ageing interventions found evidence that person-centred approaches, with behaviour change techniques such as goal setting and social interaction, ‘booster sessions’ and tailoring could optimise intervention effectiveness.^17^ We therefore hypothesised that a behaviour change intervention, Healthy Conversation Skills, could improve the PA levels and diet quality of community-dwelling older adults.

Healthy Conversation Skills was designed to provide an effective way for healthcare providers to opportunistically support individuals to maintain or improve their mental and physical wellbeing.^18^ It consists of asking open-ended (‘open discovery’) questions, reflecting on practice, listening more than giving information and supporting SMARTER (Specific, Measurable, Action-oriented, Realistic, Timed, Evaluated, Reviewed) goal setting. While Healthy Conversation Skills has been shown to be applicable, feasible and acceptable in a wide range of situations within healthcare settings,^18,19^ to the authors’ knowledge, there is no published evidence that it has been trialled in an older cohort. We therefore undertook a study of Healthy Conversation Skills in the Nutrition and Physical Activity (NAPA) Study, comprising participants of the original Hertfordshire Cohort Study (HCS) (please see the section ‘Methods’ for further study information). The NAPA study assessed the use of Healthy Conversation Skills as part of a telephone-based intervention. One aim of the study was to collect data that might inform future power calculations for a definitive study.

## Methods

### Recruitment

Participants were recruited from the HCS,^20^ which was originally established in the late 1990s to examine the relationship between growth in early life and adult diseases including cardiovascular and musculoskeletal conditions. The study design has been previously described in detail.^20^ In brief, participants were born between 1931 and 1939 and were still currently living in Hertfordshire at the time of study baseline data collection in 1998 to 2004. A total of 3225 participants were recruited to participate in a home interview with a trained research nurse, and 2997 participants (93%) subsequently attended a clinic for further physiological investigations.

**Figure 1.**
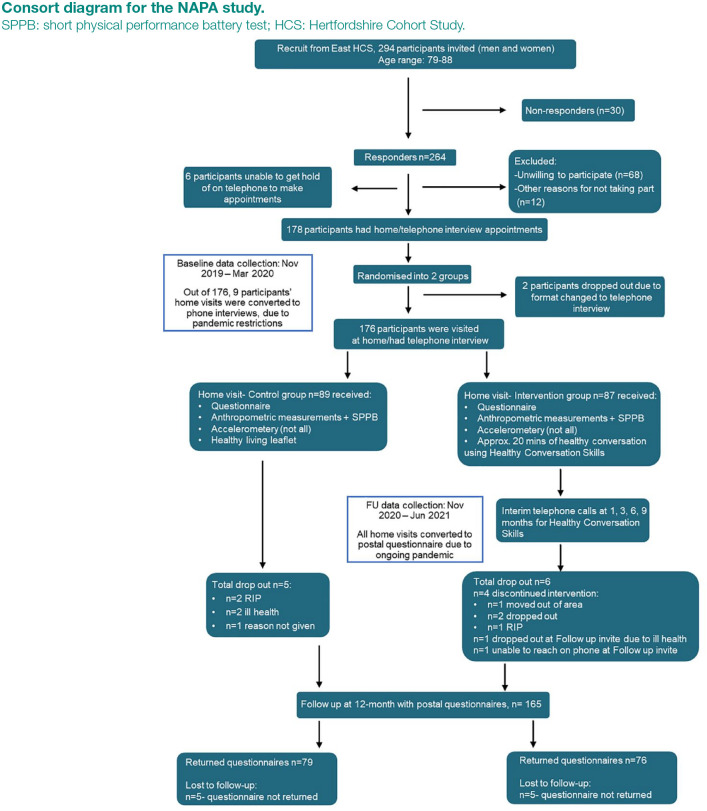
Consort diagram for the NAPA study. SPPB: short physical performance battery test; HCS: Hertfordshire Cohort Study.

Since the initial recruitment of the cohort group, there have been different follow-up studies.^20^ Of those who participated in the latter phases of the HCS, 294 participants who were still living in the county of Hertfordshire and who had participated in previous musculoskeletal waves of the cohort study were eligible for recruitment to the NAPA study. We sent invitations to this group; 264 responded as willing to participate in this phase of study. In total, 176 participants completed baseline data collection, having been randomised into either control (n = 89) or intervention (n = 87) groups by a random allocation process, as detailed in Figure 1. The study follows a randomised controlled trial (RCT) design. A pragmatic approach to randomisation was used, matching by sex. The random allocation process involved participants being divided into two groups: women and men, then each participant was allocated to one of the following groups in a rolling fashion by a third member of the research team, using a Microsoft Excel spreadsheet: Researcher 1 Control or Researcher 1 Intervention or Researcher 2 Control or Researcher 2 Intervention. Researcher 1 and Researcher 2 were the two researchers who conducted the home visits.

Ethical approval for this study was granted by the East of England–Cambridgeshire and Hertfordshire Research Ethics Committee (reference no. 11/EE/0196). Participants provided written informed consent before taking part in the study.

### Data collection

#### Questionnaire

Questionnaire data included diet and nutrition (24-item food frequency questionnaire (FFQ)^21^ and Simplified Nutritional Appetite Questionnaire (SNAQ)),^22^ PA (Longitudinal Aging Study Amsterdam Physical Activity Questionnaire (LAPAQ)),^23^ physical function (Short Form-36 physical functioning scale (SF-36 PF))^24^ and environmental and psychosocial factors, such as the 6-item Lubben Social Network Scale (LSNS-6).^25^

#### Physical measurements

Physical measurements included height, weight, grip strength and gait speed. Height was measured with the participant standing using a pocket stadiometer and was repeated three times to the nearest 0.1 cm. The weighing scales were placed on a level hard surface and participants were weighed once, without shoes and heavy items of clothing, with weight recorded to the nearest 0.1 kg. Grip strength was measured via a JAMAR dynamometer using a standard protocol.^26^ Gait speed was measured twice by timing how long it took the participant to walk 2.44 m (8 feet).

#### Accelerometery data

A smaller randomly selected sub-sample of the participants from the control and intervention groups were given accelerometers to wear at the conclusion of the home visit; a GENEActiv accelerometer (ActivInsights Limited, Cambridge, UK) worn on the dominant wrist over a 7-day period. The participants were asked to post the accelerometers back to the research centre using a pre-paid envelope.

The whole home visit took approximately 1 h and 30 min. Baseline data collection of the NAPA study commenced in mid-November 2019 and finished by late March 2020, and the 1-year follow-up occurred between November 2020 and June 2021. Due to COVID-19 restrictions arising during baseline data collection, nine participants had their baseline home visits changed to telephone interviews, and the planned 1-year follow-up home visits were converted to postal questionnaires. Therefore, at 1-year follow-up, it was not possible to repeat the physical measurements and accelerometery that were performed at the baseline home visits.

Figure 1 shows the recruitment flow of the NAPA study from baseline through to the 1-year follow-up, including details on participants who dropped out of the study. Analyses were based on the 155 questionnaires returned.

### Healthy Conversation Skills intervention

At baseline, following the questionnaire and physical measurements, the participants in the intervention group were engaged in an approximately 15- to 20-min healthy conversation regarding their lifestyle behaviours, specifically focusing on diet and PA. We used conversation prompts, mainly adopting open-ended questions related to diet and exercise. This conversation was audio-recorded with the participants’ permission. Those participants who did not give permission for the conversation to be audio-recorded were still able to continue to participate in the study. Participants in the control group were given a healthy living leaflet at the end of the home visits.

Those in the intervention group received 1-, 3-, 6- and 9-month follow-up telephone calls during which the researcher engaged the participant in healthy conversations about their lifestyle behaviours. At baseline, these conversations had aimed to prompt a lifestyle change in any area that the participant wished to focus on, such as improving their balance or eating a ‘healthier’ diet (e.g. increasing fruit and vegetable intake). The topic and areas to change were driven by the participant; if the participant did not feel there was the need for a change, then the interviewer explored and encouraged maintenance of their current routine. In the follow-up phone calls, the healthy conversations focused on reviewing progress and exploring possible future changes.

#### Outcome measures

The primary outcome measures were self-reported PA as assessed by the LAPAQ and prudent diet score. Diet was assessed using a short FFQ, which was developed to assess diet quality in older adults.^21^ A prudent diet score was calculated for each participant and was used as an indicator of diet quality. Higher prudent diet scores indicate diets characterised by more frequent consumption of fruit, vegetables, wholegrain cereals and oily fish but lower consumption of white bread, added sugar, full-fat dairy products, chips and processed meat.

There were several secondary outcome measures including scores for appetite (SNAQ); SF-36 physical function; general self-efficacy; and social network (LSNS-6).

As the LAPAQ section of the questionnaire included missing data for a proportion of participants, to maintain maximum sample study size we used a shortened version of LAPAQ, where only three domains were included: walking, cycling and sports, in accordance with the previous literature.^27^ The domains of gardening and housework were not included.

### Statistical analyses

Depending on distribution of data, baseline participant characteristics were described using mean values and standard deviations (SDs), medians and inter-quartile ranges (IQR), and frequency and percentage distributions. Differences in baseline characteristics between intervention and control groups were assessed using chi-square tests, *t*-tests and Wilcoxon rank-sum tests, as appropriate, among the entire sample and among men and women separately.

The study duration from baseline to follow-up differed between participants and ranged from approximately 12–18 months. Therefore, changes in the following scores from baseline to follow-up were annualised and used as trial outcomes: shortened LAPAQ, prudent diet, SNAQ, SF-36 physical function, general self-efficacy and LSNS-6. Normality of these outcomes was confirmed through visual inspection of histograms. Among the pooled sample of men and women, differences in outcomes between the intervention and control groups were examined using linear regression with adjustment for sex; these differences were also examined among men and women separately using simple linear regression, equivalent to performing sex-specific *t*-tests.

Analyses were conducted using Stata, release 17.0 (StataCorp, College Station, Texas, USA); *p* < 0.05 was regarded as statistically significant. Accelerometery data were processed in R (http://www.cran.r-project.org) using R-package GGIR, version 1.11-0, and methods and results have been previously published.^28^

## Results

### Baseline participant characteristics

Baseline participant characteristics of the analysis sample are presented in Table 1. Among the 155 participants who were included in the analysis sample, median (lower quartile, upper quartile) age was 83.1 (81.5, 85.5) years. Mean (SD) body mass index (BMI) was 27.2 (4.0) kg/m^2^ and 57 (37.0%) participants were ex/current smokers. There were statistically significant differences (*p* < 0.05) in the distribution of number of comorbidities between the trial arms among the entire sample and in the distribution of SF-36 physical function scores between the trial arms among men; no other baseline characteristics differed significantly between the intervention and control groups in sex-pooled or sex-stratified analyses.

**Table 1.** Baseline descriptive statistics for the 155 participants who took part in the baseline and follow-up stages.

Characteristic	Mean (SD); Median (lower quartile, upper quartile); N (%) according to characteristic and distribution of variable					
	Control			Intervention		
	All (*n* = 79)	Men (*n* = 43)	Women (*n* = 36)	All (*n* = 76)	Men (*n* = 39)	Women (*n* = 37)
Age (years)	83.4 (81.7, 86.0)	83.6 (81.7, 86.3)	83.3 (81.7, 85.5)	82.8 (81.3, 85.0)	82.3 (81.3, 84.7)	84.2 (81.2, 86.1)
Height (cm)	164.8 (8.8)	171.0 (5.0)	157.3 (6.1)	164.7 (9.7)	171.6 (6.5)	157.2 (6.4)
Weight (kg)	74.3 (14.3)	81.8 (10.3)	65.4 (13.3)	73.8 (12.7)	80.0 (10.3)	67.6 (11.9)
BMI (kg/m^2^)	27.3 (4.2)	28.0 (3.7)	26.3 (4.7)	27.1 (3.9)	26.9 (3.0)	27.2 (4.6)
Ever smoked	32 (41.0%)	21 (48.8%)	11 (31.4%)	25 (32.9%)	15 (38.5%)	10 (27.0%)
Alcohol (units per week)	1.6 (0.0, 6.6)	1.7 (0.1, 9.1)	0.8 (0.0, 5.5)	3.3 (0.2, 8.4)	4.5 (0.6, 8.6)	1.8 (0.0, 7.0)
Social class (manual)	45 (58.4%)	24 (58.5%)	21 (58.3%)	38 (52.8%)	20 (57.1%)	18 (48.6%)
Live alone	26 (32.9%)	8 (18.6%)	18 (50.0%)	25 (32.9%)	12 (30.8%)	13 (35.1%)
Grip strength (kg)	27.6 (8.7)	33.4 (6.6)	21.0 (5.4)	27.0 (9.7)	34.1 (7.5)	19.9 (5.4)
Gait speed (m/s)	0.61 (0.18)	0.62 (0.18)	0.61 (0.17)	0.65 (0.19)	0.70 (0.19)	0.60 (0.18)
EuroQoL anxiety/depression	18 (23.1%)	9 (21.4%)	9 (25.0%)	19 (25.0%)	8 (20.5%)	11 (29.7%)
Number of medications	4.0 (2.0, 7.0)	5.0 (2.0, 8.0)	4.0 (2.5, 6.0)	5.0 (3.0, 7.0)	5.0 (3.0, 7.0)	5.0 (4.0, 7.0)
Number of comorbidities^a^	2.0 (1.0, 2.0)	2.0 (1.0, 2.0)	1.5 (1.0, 3.0)	2.0 (1.5, 3.0)	2.0 (2.0, 3.0)	2.0 (1.0, 3.0)
Shortened LAPAQ score	30.0 (15.0, 60.0)	30.0 (10.0, 60.0)	32.1 (18.6, 70.0)	30.0 (14.6, 60.0)	30.0 (15.0, 60.0)	30.0 (14.3, 60.0)
Prudent diet score	0.0 (1.6)	-0.4 (1.3)	0.5 (1.7)	0.1 (1.3)	0.0 (1.3)	0.2 (1.3)
SNAQ score	16.0 (15.0, 17.0)	16.0 (15.0, 17.0)	16.0 (15.0, 16.0)	15.0 (14.0, 16.0)	16.0 (15.0, 17.0)	15.0 (14.0, 16.0)
SF-36 physical function score^b^	80.0 (50.0, 90.0)	70.0 (50.0, 90.0)	80.0 (50.0, 90.0)	75.0 (57.5, 95.0)	90.0 (70.0, 95.0)	65.0 (50.0, 85.0)
General self-efficacy score	15.0 (14.0, 16.0)	15.0 (14.0, 16.0)	15.0 (14.0, 16.0)	15.0 (14.0, 15.0)	15.0 (14.0, 15.0)	15.0 (14.0, 15.0)
LSNS-6 score	17.0 (5.6)	16.6 (5.7)	17.6 (5.6)	16.6 (5.3)	16.4 (5.3)	16.9 (5.3)

LAPAQ: Longitudinal Aging Study Amsterdam Physical Activity Questionnaire; shortened version only included walking, cycling and sports domains; EuroQoL anxiety/depression: moderate or extremely anxious/depressed; SNAQ: Simplified Nutritional Appetite Questionnaire; SF-36: Short Form 36 Health Survey Questionnaire; LSNS-6: Lubben Social Network Scale; SD: Standard deviation.

a Statistically significant difference (*p* < 0.05) between control and intervention groups among the combined sample of men and women.

b Statistically significant difference (*p* < 0.05) between control and intervention groups among men.

In the intervention group, 4/87 participants did not fully complete the intervention, a 4.6% drop-out rate. At 1-year follow-up, in total, 11/176 participants who were visited at baseline had dropped out, including the four who did not complete the intervention, a 6.3% drop-out rate. Of the remaining 165 participants who were contacted at follow-up through postal questionnaires, 10 questionnaires were not returned, resulting in a sample of 155 participants who returned the questionnaires at the 1-year follow-up.

### Differences in outcomes between trial arms

Differences in annual changes in outcomes between the control and intervention groups are presented in Table 2. In sex-pooled analyses, no statistically significant differences in outcomes between the trial arms were observed (*p* > 0.07 for all associations). There were also no significant differences for men or women in the intervention group compared with the control group. However, among women, there was a trend for greater improvement in prudent diet scores in the intervention group (0.5 (−0.1, 1.1), *p* = 0.075) and among men, there was a trend for reduced decline in SF-36 physical function scores in the intervention group (6.9 (−0.9, 14.7), *p* = 0.081).

**Table 2.** Differences in annual changes in outcomes from baseline to 1-year follow-up between trial arms.

Outcomes	Pooled and adjusted for sex		Men		Women	
	Estimate (95% CI)	*p*	Estimate (95% CI)	*p*	Estimate (95% CI)	*p*
*Primary outcomes*						
Shortened LAPAQ	3.4 (-15.4, 22.1)	0.724	6.7 (-22.7, 36.1)	0.649	-1.0 (-22.2, 20.3)	0.928
Prudent diet score	0.4 (-0.1, 1.0)	0.132	0.4 (-0.6, 1.3)	0.445	0.5 (-0.1, 1.1)	0.075
*Secondary outcomes*						
SNAQ score	0.3 (-0.2, 0.8)	0.299	0.0 (-0.7, 0.7)	0.918	0.6 (-0.2, 1.4)	0.117
SF-36 physical function score	4.5 (-0.4, 9.4)	0.072	6.9 (-0.9, 14.7)	0.081	1.7 (-4.0, 7.4)	0.549
General self-efficacy score	0.5 (-0.2, 1.2)	0.176	0.5 (-0.4, 1.5)	0.270	0.5 (-0.7, 1.6)	0.418
LSNS-6 score	0.2 (-1.4, 1.8)	0.799	-0.3 (-2.8, 2.2)	0.821	0.8 (-1.2, 2.8)	0.444

LAPAQ: Longitudinal Aging Study Amsterdam Physical Activity Questionnaire; shortened version only included walking, cycling and sports domains; SNAQ: Simplified Nutritional Appetite Questionnaire; SF-36: Short Form 36 Health Survey Questionnaire; LSNS-6: Lubben Social Network Scale; SD: standard deviation. Differences in annual changes from baseline to follow-up between the control and intervention groups were estimated using linear regression; positive estimates reflect greater longitudinal increases (or reduced declines) in the intervention group compared to the control group; negative estimates reflect reduced increases (or greater declines) in the intervention group.

## Discussion

We present findings from a study of a behavioural intervention, Healthy Conversation Skills, in a cohort of older adults aged ⩾79 years. For the first time, we have shown that it is viable to deliver Healthy Conversation Skills remotely (via telephone) to an older population. In total, only 4.6% of the participants failed to complete the intervention. This is important as it suggests it is possible to engage with older adults by telephone in their own homes to have behaviour change conversations, even during the COVID-19 pandemic.

We did not observe any significant changes in PA or other outcomes, as a result of the intervention, but acknowledged from the outset that the study was not designed to be powered to show a definitive effect. In addition, the COVID-19 pandemic restrictions have been particularly severe in this age group due to the high prevalence of shielding, and this may have had a significant impact upon the adaptations that these participants could make. The direct impact of the pandemic on older adults from the HCS cohort has been previously described elsewhere;^29^ 47% of respondents reported being less physically active compared to pre-pandemic times, with only 5% reporting being more physically active. This has also been observed elsewhere.^30–33^ By contrast, although diet quality remained largely unchanged during the pandemic in the HCS cohort, almost a fifth of participants reported eating less than before the pandemic,^29^ again consistent with other studies.^30^

A key positive feature of our study was the high completion rate. A lack of compliance has proved to be a common barrier in interventions designed to improve PA levels, physical function or nutrition-related outcomes in older adults. Drop-out rates appear to be affected by the type of PA intervention trialled. Integrative approaches, which commonly attempt to embed functional exercises into daily life (e.g. climbing the stairs), typically perform better at retaining participants in comparison to structured training and combination programmes, like exercise classes.^34^ In our study, the drop-out rate in the intervention arm was lower than many studies, which typically report figures of 5%–29%.^34^ A systematic review of RCTs that investigated nutrition interventions with community-dwelling older adults, including mostly nutrition education and counselling interventions (*n* = 10), found follow-up rates of 70%–100% (on average 85%).^35^

The use of Healthy Conversation Skills in the NAPA Study is likely to have differed compared with previous studies that have utilised Healthy Conversation Skills. To date, the impact of the Healthy Conversation Skills intervention has only been evaluated in younger populations.^18,19,36,37^ The influential factors that affect PA participation and dietary choices are likely to be different for the various age groups.^38,39^ For instance, the psychological mind-set of an older adult is likely to differ from a pregnant woman or a parent. Those in the younger spectrum of the lifecourse may focus more on future proofing as their greater outcome expectancy (i.e. the belief that implementing a behavioural change may indeed produce health benefits in the future) may incentivise behaviour change more than for those in the latter aspect of the lifecourse, whose future is inevitably likely to be shorter.^40^ Our finding of a high engagement rate indicates the versatility of Healthy Conversation Skills within the cohort of older adults. One example of a difference in the approach used when compared with previous studies was the modification on the avoidance of usage of terms ‘goal’ and ‘goal setting’ which was made early in the study. The implementation, mechanism and usage of the intervention have been assessed in a process evaluation using the Medical Research Council (MRC) guidance, which has been described separately as Supplementary Material (see Supplementary File 1).

### Strengths and limitations

A strength of this study was the RCT design used, including the random allocation of participants to trial arms. Nonetheless, at baseline, participants in the intervention group had significantly greater numbers of comorbidities. Study participants were all white British and in their 80s; therefore, we cannot claim that these results are generalisable to other populations of community-dwelling older people. The researchers that conducted the baseline data collection also delivered the intervention and thus could not be blinded to the intervention status of participants. However, follow-up data were collected remotely via post therefore removing researcher bias. Although the trial outcome measures were based on self-reported data, the measures used to assess PA (shortened LAPAQ), diet quality (prudent diet score), appetite (SNAQ score), physical function (SF-36 physical function score) and social network (LSNS-6 score) have been shown to yield useful information among older populations.^21–25,27^ Given the advent of the COVID-19 pandemic shortly after the baseline data collection, it was not possible to perform home visits at the 1-year follow-up; instead, participants were followed up via postal questionnaire, and therefore, it was not possible to repeat the physical measurements. There was a considerable amount of missing data for some of the study outcomes at follow-up, which reduced the sample size that could be used for analyses. The value of this study is that it is the first to test the suitability of using Healthy Conversation Skills with an older community-dwelling population. Overall, the research team’s long-standing relationship with HCS cohort members is likely to have enhanced engagement in this study, fostering rapport and trust among participants, thus also possibly promoting engagement with the Healthy Conversation Skills intervention.

## Conclusion

We have demonstrated that it is viable to use Healthy Conversation Skills in an ageing cohort and deliver an intervention remotely, via telephone. The low drop-out rate suggests this approach was acceptable to an older adult cohort. Given the shift to an ageing population, there is an imperative to find ways to support older adults to maintain good health throughout their later years. We know this can be achieved by improving dietary quality and increasing PA. Thus, further evaluation of this intervention with a larger, more diverse group of community-dwelling older people is warranted.

## Supplemental Material

sj-docx-1-rsh-10.1177_17579139241262657 – Supplemental material for A Healthy Conversation Skills intervention to support changes to physical activity and dietary behaviours in community-dwelling older adults during the COVID-19 pandemic Supplemental material, sj-docx-1-rsh-10.1177_17579139241262657 for A Healthy Conversation Skills intervention to support changes to physical activity and dietary behaviours in community-dwelling older adults during the COVID-19 pandemic by J Zhang, I Bloom, LD Westbury, G Bevilacqua, KA Ward, M Barker, W Lawrence, C Cooper and EM Dennison in Perspectives in Public Health
