# Supplementary material for: A Healthy Conversation Skills intervention to support changes to physical activity and dietary behaviours in community-dwelling older adults during the COVID-19 pandemic
Source: Perspect Public Health. 2024 Aug 1;146(2):104–12. doi: 10.1177/17579139241262657 (PMC13091919; doi:10.1177/17579139241262657)
Supplement: sj-docx-1-rsh-10.1177_17579139241262657 – Supplemental material for A Healthy Conversation Skills intervention to support changes to physical activity and dietary behaviours in community-dwelling older adults during the COVID-19 pandemic [file sj-docx-1-rsh-10.1177_17579139241262657.docx]

## **Process evaluation of the Healthy Conversation Skills intervention in the Nutrition and Physical Activity (NAPA) Study**

As part of the Nutrition and Physical Activity (NAPA) Study, we conducted a process evaluation of the behaviour change intervention that was carried out in community-living older adults. We assessed recruitment, retention and the practicality of delivering the intervention in this population and to understand what adaptations might be required. Here we summarise the details of the process evaluation.

## Methods

In total, ten participants from the NAPA study were randomly selected for the process evaluation, using the following criteria:

1. The participant must have completed the full intervention, i.e. have a Case Report Form and Healthy Conversation Skills interview recordings for all five time points. The five time points consisted of baseline home visit, 1, 3, 6, and 9-months follow-up telephone calls.

2. All participants eligible for random selection must have received their 3-month follow-up call after the announcement of the first UK COVID-19 lockdown, which occurred on 23rd March 2020. The rationale for this criterion was that as the home visits spanned from November 2019 – March 2020, some participants would have had their 3-month follow-up pre-lockdown and others post-lockdown. The criterion adopted ensured consistency and removed one possible confounding factor.

3. We aimed for an equal number of men and women, and a reasonable spread between the two researchers that carried out the field work for the study.

The process evaluation was guided by the Medical Research Council guidance ^1^, encompassing assessment of three aspects, namely the implementation of the intervention, mechanisms of its impact, and context.

A different team of researchers assessed competency in use of Healthy Conversation Skills and other aspects of the process evaluation, to increase objectivity. The assessors listened to the audio recordings from all contact points of the ten participants and used the information and notes in each participant’s Case Report Form to fill out the following two forms for each conversation: a Competency Coding Tool to assess the use of open discovery questions (ODQs) and listening (this has been used in a previous study assessing competence in using Healthy Conversation Skills ^2^), and a SMARTER coding rubric to assess overall SMARTER goal-setting, as well as each domain of the SMARTER goal. Assessors also completed a checklist for each participant, which included noting down topics discussed by the participants, behaviour change goals set, any changes made, signs of resistance to the intervention, and evidence of impact of COVID-19.

The researchers who had undertaken the fieldwork read through the notes and forms completed by the assessors. Mean Healthy Conversation Skills competency scores for each researcher were calculated. Using the completed checklists and the Case Report Forms, content analysis was carried out to identify information related to the implementation of the intervention, mechanisms of impact and context. Implementation was assessed by examining the use of Healthy Conversation Skills and any adaptations. The mechanisms of impact were assessed by noting any changes made by the participant as a result of the intervention. We monitored signs of increased self-efficacy, as we hypothesised that this would make changes in behaviour more likely. We also assessed whether there was any resistance displayed by the participants to engaging in behaviour change during the course of the intervention. In relation to context, we assessed contextual factors that may have influenced participants’ responses to the intervention, especially the impact of COVID-19 on participants’ ability to socialise, to access services or shops and to perform PA.

## Results

The process evaluation included ten participants, with an equal split between men and women. Participants had a median age of 82.3 years (IQR 81.3 - 83.1 years).

#### Implementation

To assess intervention fidelity, Healthy Conversation Skills competency scores for the researchers delivering the intervention were calculated for the subset of participants (n = 10). Researchers demonstrated a relatively high level of competence in all three skills (asking ODQs, listening and SMARTER goal-setting); total mean score for overall use of Healthy Conversation Skills was 8.2 out of a possible score of 10. The intervention delivery needed to be somewhat adapted in this population. After the first few home visits, researchers noticed that participants were not particularly receptive to the terms “goal setting” or “goals” so, on reflection and in discussion with the wider research team, including experts in Healthy Conversation Skills, these terminologies were avoided for the remainder of the study. Examples of ODQs used included “How do you feel about your lifestyle at the moment?” and “What do you think you could do…?”. In the follow-up phone calls, the healthy conversations focused on reviewing progress and exploring possible further changes or facilitating goal adjustment if applicable. Overall, a more flexible approach to the SMARTER goal setting was adopted that felt more acceptable to this cohort. It is possible that this adaptation to the intervention delivery may have impacted its efficacy.

Where relevant, for example, if participants appeared keen to maintain or prevent decline of muscle strength/function or balance, researchers explored some more specific exercises to support these aims. In some cases, researchers encouraged the participant to look online (e.g. on the NHS website) for further balance or muscle strengthening exercises, or to find information about PA. However, some participants did not have access to the internet or were not computer literate, relying on relatives to access and print off information.

#### Mechanisms of impact

One premise that underpins Healthy Conversation Skills is that if an individual increases their self-efficacy, then they are more likely to change a behaviour. As a result of having a healthy conversation, researchers identified that some participants wanted information in order to support a change they wished to make, and as a result of this, participants were prompted to take first steps, in turn appearing to increase their self-efficacy and overcome challenges. Some participants demonstrated resilience, despite adverse circumstances or facing obstacles, for example those posed by the pandemic, finding ways to engage in healthy behaviours.

*“One thing as you get older; I mean, 82 now. You do notice certain things…, strength-wise; ease off bit…and you do have a job to keep that going. But…we still try to do everything we can; that’s all we can do, really.”* Participant 2

In terms of changes made due to the intervention, most participants fully or partly achieved their goals. Some participants displayed signs of resistance to the intervention, which appeared to link with poorer attainment of goals, perhaps indicating that these had been less ‘realistic’. Different levels of readiness to change and participants’ mindset appeared was linked to likelihood of achieving own goals. Some lacked belief that anything would help with promoting muscle strength, or lacked insight into the benefits of PA.

*“...I don’t know if I can do anything… (to get over barriers to doing exercises); it’s just me...it’s quite difficult… to embark on a major change at a certain age…”* Participant 7

#### Context

The pandemic and lockdown impacted on participants’ way of life, acutely and also potentially in the long-term. There was an impact on the availability of social activities/opportunities, shopping habits and an inability to perform usual PA e.g. playing golf, dancing, exercise classes, walking groups, due to closures and cancellations related to pandemic restrictions. Increased foot traffic from other users was mentioned as an inhibitor to walking. For some, the fear of going out and the general pandemic situation appeared to have affected motivation to do exercises. Some displayed reticence to resume usual activities following lockdown, while others appeared keener to re-establish activities.

*(Asked about exercises) “...I’ll be quite honest, a lot of those sort of things have gone a bit by the board…yes, I apologise… I think if I… persevered with them…there would probably have been a result… with all that’s going on around us at the moment (start of 1st lockdown)....”* Participant 5

The pandemic appears to have had an impact on participants’ responses to the intervention, and overall, possibly served to diminish its effects.

Other contextual factors likely affected how participants responded to the intervention, affecting the degree to which participants might have engaged in making plans for behaviour change, and engaged in change. Social aspects were highlighted throughout as an integral part of activities, e.g. walking with family members or friends, social aspects of clubs and societies e.g. gardening club. Support from family with things like shopping, seeking exercise options, reminding to perform exercises. Health and illness were also important; participants spoke of impact of pain/discomfort/falls and fractures on PA e.g. osteoarthritis in hands causing pain and discomfort, preventing use of weights. There was mention of feeling tired and natural age-related decline. Bereavement or ill health of partner were important concerns. One participant was a carer for her husband, and she reinforced the importance for her to stay fit to look after her husband. Another participant had recently lost his wife before the start of the intervention and was getting used to be alone, this led to him being socially isolated during the pandemic. Local environment was important in enabling PA e.g. availability and size of garden, availability of green space/easy access walking routes. Type of PA undertaken was often weather-dependent. All these factors might affect older people's engagement in healthy behaviours and help to explain why uptake/effectiveness of the intervention may have varied.

## Discussion and Conclusion

In future studies, it might be helpful to set out the expectations and ambitions of the study which is to support change by way of supporting participants to set goals. It could be necessary for researchers/practitioners to be more specific at the start of the healthy conversation that this will focus on possible changes to diet/ PA and more explicit about supporting achievement of changes to behaviour. With an ageing population, over time there will be a degree of physical decline, therefore *maintenance* of health behaviours/physical capability should be viewed as a valuable goal in itself.

Some participants displayed signs of resistance to the intervention. For example, some appeared to lack insight on the benefits of PA at this age. Within the context of supporting PA, the promotion of bone and muscle health and balance appeared to be a novel concept for some participants, which is consistent with other studies. These have indicated a lack of awareness among community-living adults about PA guidelines, particularly those regarding muscle strength ^3^, or a lack of insight into dietary recommendations specific to older adults, compared to those for the general population, and the links between nutrition and health in older age ^4, 5^. This is of concern given the important role of knowledge or awareness among older adults, for example, of the current government recommendations and of the health benefits of being active and eating a healthier diet, for engaging in healthier behaviours ^6-8^. Regarding other signs of resistance to the intervention, some participants appeared to have accepted that there was nothing that could be done regarding any perceived age-related decline or did not believe that their muscle strength could be improved. Others have also found a sense of resignation to age-related decline in physical ability among some community-living older people ^9^. Thus, in our study there appeared to be different outcome expectancies, that is, the belief that a behaviour will lead to a desired outcome ^10^, with some believing that changing unhealthy behaviours would not necessarily improve their health, whereas others believed that PA and/or a healthy diet would keep them healthy and independent. In this way, outcome expectancies appeared to influence PA and diet-related behaviours in these older adults. Social Cognitive Theory asserts the importance of psychological factors such as self-efficacy and outcome expectancies for an individual’s behaviour; thus, if an individual believes that they can undertake an action (self-efficacy) and that this will lead to a positive outcome (outcome expectancy), they will be more likely to overcome barriers to health-promoting behaviours and to make a change to their behaviour ^11^. All this suggests that there is a need to raise awareness among older adults, and also their family members or carers, of the importance of nutrition and PA for healthy ageing, including older adults’ particular needs, e.g. higher protein requirements, weight loss only recommended in specific situations, regular targeted activities that improve or maintain balance and muscle strength, in addition to more general PA ^4, 5, 9^. As such, in addition to increasing self-efficacy and motivation to overcome barriers to healthier behaviours and to making changes, information could be integral to a healthy conversation, i.e. exploring what participants know, what they would like to know, how will that translate into action. In some cases, it could be necessary to include a more systematic explanation about benefits of PA/diet for health in later life, to facilitate further conversations. Separately, broader public health interventions appear to be necessary to raise wider awareness about the benefits of PA and diet for health, even at older ages.

Findings suggested some positive impacts on self-efficacy and suggested that some older adults who might already be self-motivated, ‘active’ or already have ‘healthy’ diets could still benefit from ‘light-touch’ interventions. Depending on an individual’s ‘readiness to change’ their behaviour, i.e. an individual's ‘stage of change’, this is likely to impact their level of engagement with the intervention and degree of attainment of behaviour change goals ^12, 13^.

For some participants, there were issues of digital literacy/access, with some not having access to the internet, or not feeling confident to use a computer or go online, needing family members to access online resources on their behalf. Thus, the digital literacy of participants could be an important consideration in the design of a future study. Other contextual factors are also likely to have affected how participants responded to the intervention, for example, their overall health and physical capability, any exacerbation of a health condition, a recent bereavement or being socially isolated. Consideration of these factors might help to explain why uptake or effectiveness such an intervention may vary among older people.

The process evaluation findings suggested that the intervention was feasible and acceptable to participants; some participants displayed signs of resistance to engaging in behaviour change. The COVID-19 pandemic and other factors (e.g. health status) affected participants’ response to the intervention. Further evaluation of the intervention should consider aspects identified in the process evaluation, including adaptations that could be beneficial to optimise the intervention in this population. Intervention delivery will need take account of issues that are likely to affect the engagement of older individuals in healthy behaviours (e.g. social isolation, ill health/bereavement).

## References

1. Moore GF, Audrey S, Barker M, et al. Process evaluation of complex interventions: Medical Research Council guidance. *Br Med J* 2015; 350: h1258. 10.1136/bmj.h1258 2015/03/21. DOI: 10.1136/bmj.h1258.

2. Lawrence W, Vogel C, Strömmer S, et al. How can we best use opportunities provided by routine maternity care to engage women in improving their diets and health? *Maternal & Child Nutrition* 2020; 16: e12900. DOI: <https://doi.org/10.1111/mcn.12900>.

3. Gluchowski A, Bilsborough H, McDermott J, et al. 'A Lot of People Just Go for Walks, and Don't Do Anything Else': Older Adults in the UK Are Not Aware of the Strength Component Embedded in the Chief Medical Officers' Physical Activity Guidelines - A Qualitative Study. *Int J Environ Res Public Health* 2022; 19. DOI: 10.3390/ijerph191610002.

4. Browne S, Geraghty A and Corish C. Advances in knowledge of screening practices and their use in clinical practice to prevent malnutrition. *Proc Nutr Soc* 2022; 81: 41-48. DOI: 10.1017/S0029665121003670.

5. Castro PD, Reynolds CME, Kennelly S, et al. An investigation of community-dwelling older adults' opinions about their nutritional needs and risk of malnutrition; a scoping review. *Hum Nutr Clin Nutr* 2021; 40: 2936-2945. DOI: 10.1016/j.clnu.2020.12.024.

6. Appleton KM, McGill R, Neville C, et al. Barriers to increasing fruit and vegetable intakes in the older population of Northern Ireland: low levels of liking and low awareness of current recommendations. *Public Health Nutr* 2009; 13: 514-521. DOI: 10.1017/S1368980009991790.

7. Stroebele-Benschop N, Depa J and de Castro JM. Environmental Strategies to Promote Food Intake in Older Adults: A Narrative Review. *J Nutr Gerontol Geriatr* 2016; 35: 95-112. DOI: 10.1080/21551197.2016.1173614.

8. Boulton ER, Horne M and Todd C. Multiple influences on participating in physical activity in older age: Developing a social ecological approach. *Health Expect* 2018; 21: 239-248. DOI: <https://doi.org/10.1111/hex.12608>.

9. Payne L, Harris P, Ghio D, et al. Beliefs about inevitable decline among home-living older adults at risk of malnutrition: a qualitative study. *J Hum Nutr Diet* 2020; 33: 841-851. DOI: <https://doi.org/10.1111/jhn.12807>.

10. Grembowski D, Patrick D, Diehr P, et al. Self-efficacy and health behavior among older adults. *Journal of health and social behavior* 1993; 34: 89-104.

11. Purdie N and McCrindle A. Self-Regulation, self-efficacy and health behavior change in older adults. *Educ Gerontol* 2002; 28: 379-400. DOI: 10.1080/03601270290081353.

12. Jiménez-Zazo F, Romero-Blanco C, Castro-Lemus N, et al. Transtheoretical Model for Physical Activity in Older Adults: Systematic Review. *Int J Environ Res Public Health* 2020; 17. DOI: 10.3390/ijerph17249262.

13. Prochaska JO and DiClemente CC. Stages and processes of self-change of smoking: toward an integrative model of change. *J Consult Clin Psychol* 1983; 51: 390-395.
